# Tumor metabolism rewiring in epithelial ovarian cancer

**DOI:** 10.1186/s13048-023-01196-0

**Published:** 2023-06-05

**Authors:** Ming Wang, Jingjing Zhang, Yumei Wu

**Affiliations:** Department of Gynecologic Oncology, Beijing Obstetrics and Gynecology Hospital, Capital Medical University, Beijing Maternal and Child Health Care Hospital, 17 Qihelou St, Dongcheng District, Beijing, 100006 China

## Abstract

The mortality rate of epithelial ovarian cancer (EOC) remains the first in malignant tumors of the female reproductive system. The characteristics of rapid proliferation, extensive implanted metastasis, and treatment resistance of cancer cells require an extensive metabolism rewiring during the progression of cancer development. EOC cells satisfy their rapid proliferation through the rewiring of perception, uptake, utilization, and regulation of glucose, lipids, and amino acids. Further, complete implanted metastasis by acquiring a superior advantage in microenvironment nutrients competing. Lastly, success evolves under the treatment stress of chemotherapy and targets therapy. Understanding the above metabolic characteristics of EOCs helps to find new methods of its treatment.

## Background

EOC accounts for about 2.5% of all female malignant tumors, but its mortality rate ranks the first in malignant tumors of the female reproductive system. During the past two decades, the overall mortality rate of all cancers decreased by about 29% with the advancement of screening and treatment modalities [1]. However, the 5-year survival rate of ovarian cancer remained unchanged, only about 48.6% [2]. Currently, debulking surgery combined with adjuvant chemotherapy and/or targeted therapy is the standard treatment of EOC [3]. Targeted therapies of EOC include vascular endothelial growth factor (VEGF) inhibitors [4], poly ADP-ribose polymerase inhibitors (PARPi) [5, 6], and immune checkpoint inhibitors [7]. However, many patients did not benefit from the targeted treatments for the absence of related gene mutations. For example, the overall germline and somatic mutation rates of *BRCA1/2* or homologous recombination deficiency in EOC patients are only about 24% [8]. Immune checkpoint inhibitors enable T cells to kill tumors by reversing the combination of programmed cell death protein-1 (PD-1) of T cells and programmed cell death-Ligand 1(PD-L1) of tumor cells which is aimed at patients with recurrent EOC characterized by microsatellite instability-high, mismatch repair deficiency, or high tumor mutation burden [8]. However, EOC lacks T lymphocyte infiltration and the infiltrating T lymphocytes cannot recognize all tumor antigens which results in poor response rates of immunotherapy [9]. After standard debulking surgery and systemic chemotherapy combined with VEGF inhibitors or PARPi, the relapse was inevitable in more than 80% of patients of Stage III-IV EOC patients [2]. Therefore, it is urgent to further clarify the biological behavior of EOC cells to find new treatment methods.

Carcinogenesis is a multistep process and needs a set of functional capabilities which include sustaining proliferative signaling, evading growth suppressors, resisting cell death, enabling replicative immortality, inducing/accessing vasculature, activating invasion and metastasis, reprogramming cellular metabolism, and avoiding immune destruction [10]. Of these hallmarks, reprogramming cellular metabolism provides the bases for uncontrolled sustaining cell proliferation, activating invasion and metastasis, and avoiding immune destruction which is also the characteristics of EOC. However, different cancers have their genotypes and the same genotype may have discrete phenotypes for disrupted differentiation, epigenetic reprogramming, varying origins, and specific microenvironment. High-grade serous ovarian carcinoma (HGSOC) has its specific origins, invasion, metastasis, and treatment response. The HGSOC mainly originated from fallopian [11], which remains only EOC and serous tubular intra-epithelial carcinomas in most cases [12]. Different from many cancers which require the blood or lymph to metastasize, HGSOC grows on the surface of the ovary or fallopian tube and typically spreads by direct extension to the adjacent organs within the peritoneal cavity. Once the cells can implant and seed distant organs or tissues with nests of cancer cells, they develop rapidly into secondary tumor nodules, such as omentum which was mainly compromised with adipocytes. Besides, p53 plays dual roles in tumor responses to chemotherapeutic responses in different tumors [13]. P53 is the single most frequently altered gene in human cancers and is present in approximately 50% of all invasive tumors [14]. P53 mutation predicted resistance to chemotherapy in diffuse large B-cell lymphoma, esophageal cancer, and oropharyngeal cancers [15]. The mechanisms involved enhancing drug efflux and metabolism, promoting survival, inhibiting apoptosis, upregulating DNA repair, suppressing autophagy, elevating microenvironmental resistance, and inducing a stem-like phenotype. Whereas, head and neck squamous cancer, some breast cancer, and ovarian cancers were associated with sensitivity to certain chemotherapeutic agents [16]. In locally advanced breast cancer, P53 mutated non-inflammatory carcinomas had a high rate of complete pathological response to dose-dense doxorubicin-cyclophosphamide chemotherapy, while p53 wild-type tumors never achieved complete response [17]. Besides, the rapid proliferation of EOC cells needs a highly efficient power on the perception, uptake, utilization, and regulation of glucose, lipids, and amino acids. Further, tumor cells complete implanted metastasis by acquiring a superior advantage in microenvironment nutrients competing. Lastly, tumor cells succeed evolve under the treatment stress of chemotherapy and target therapy.

Therefore, we summarize the metabolic rewiring of HGSOC cells to meet the uncontrolled sustain proliferation, implanted metastasis, and evolution under treatment stress to ultimately provide new strategies for its treatment.

## Metabolism alteration to sustain uncontrolled proliferation in ovarian cancer cells

Rapid proliferation is the first hallmark of HGSOC cells which need a large amount of energy supply and carbon to rebuild biomasses, such as bio-membrane, nucleic acids, and proteins. Reprogramming of glucose, lipids, and amino acids is the well-known hallmark of tumor metabolism which provide the basis of its proliferation [10].

### Different energy supply modalities of HGSOC

**Fig. 1:**
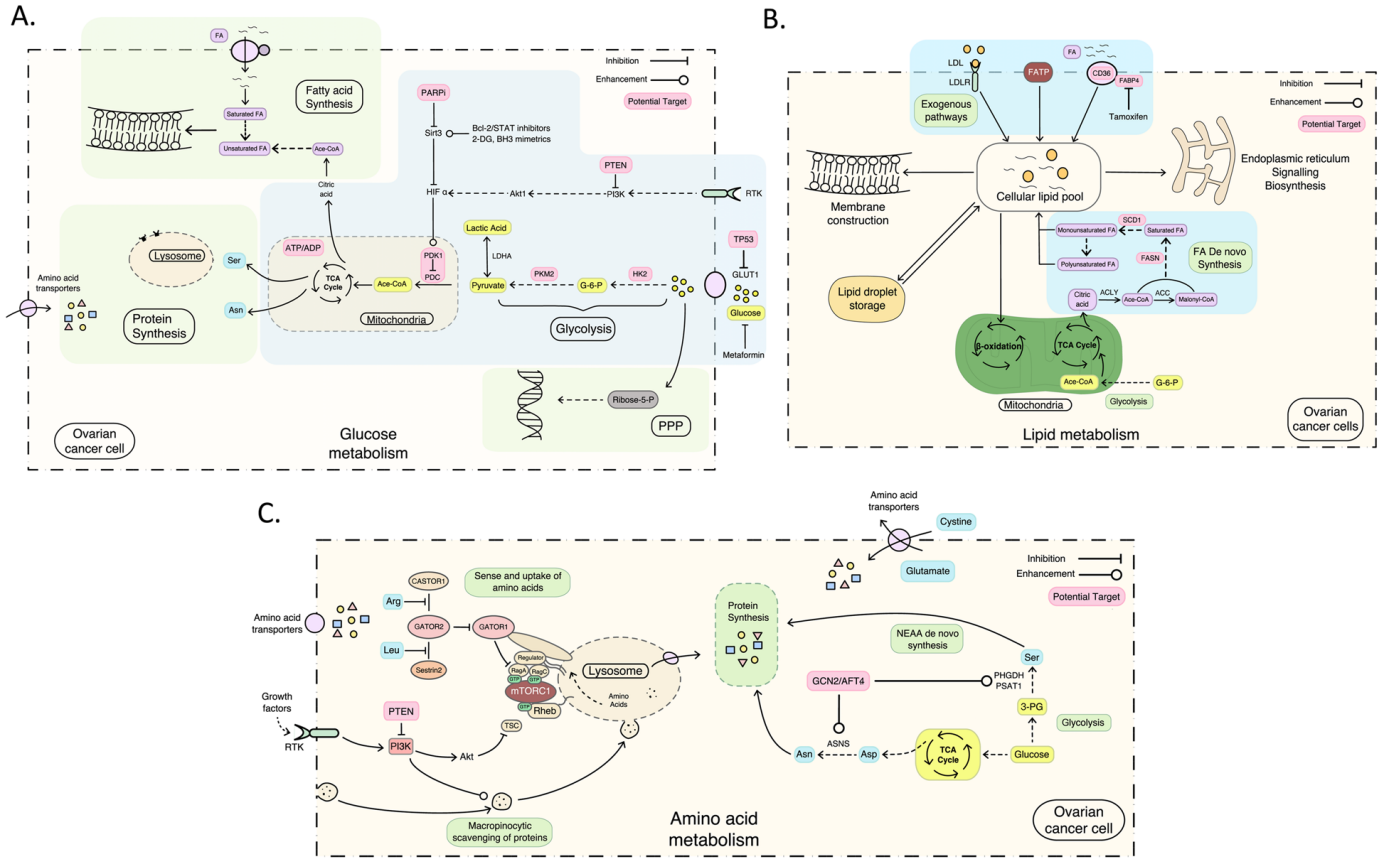
Metabolism rewiring of ovarian cancer cells. **A** shows the glucose metabolism alterations; (B) shows the lipid metabolism alterations; **C** shows the amino acid metabolism alterations. PDK-1, pyruvate dehydrogenase kinase-1; PDC, pyruvate dehydrogenase complex; KM2, pyruvate kinase M2; GLUT1, glucose transporter 1; LDHA, lactate dehydrogenase A; RTKs, receptor tyrosine kinases; FAs, fatty acids; PI3K, phosphoinositide 3-kinase; FASN, fatty acid synthase; SCDs, stearoyl CoA desaturases; mTORC1, mechanistic target of rapamycin complex 1; NEAAs, non-essential amino acids; TCA cycle, tricarboxylic acid cycle

The first feature of metabolism in malignant tumor cells is the distinct glucose metabolic mode from normal cells (see Fig. 1A). The pathways providing energy in the form of adenosine triphosphate are glycolysis and oxidative phosphorylation (OXPHOS). Normal cells obtain energy generally through OXPHOS when oxygen is sufficient. While insufficient, energy is mainly supplied by glycolysis. Unlike normal cells which obtain energy through mitochondrial respiration, tumor cells prefer to use glycolysis not OXPHOS to obtain energy, even when oxygen and glucose are sufficient, known as the Warburg effect or aerobic glycolysis [18]. Warburg's effect gives the cancer cells a survival advantage in the hypoxic tumor microenvironment (TME) and protects them from cytotoxic effects of oxidative damage and apoptosis. In the process of glycolysis, the activity of glycolytic enzymes is inhibited with lactic acidosis and decreased glucose supply. Cancer cells improve the utilization efficiency of glucose by regulating glucose aerobic oxidation and transitioning to OXPHOS to maintain cell growth and proliferation [19–21], which shows dual metabolic characteristics of glycolysis and non-glycolysis phenotypes.

Pyruvate dehydrogenase kinase (PDK) isoforms 1–4 and pyruvate dehydrogenase complex (PDC) regulate the metabolic shift between aerobic glycolysis and OXPHOS. PDC is a gatekeeper of glucose oxidation which converts pyruvate to acetyl-CoA and bridges glycolysis with the Krebs cycle [22]. The gatekeeper is regulated by phosphorylation (PDKs) and dephosphorylation (pyruvate dehydrogenase phosphatases). PDK is an enzyme that inhibits PDC catalyzing the decarboxylation of pyruvate to acetyl-CoA by phosphorylation [23]. In the cancer cells, hypoxia, and dysregulate signals can enhance glycolysis or hinder OXPHOS through the imbalance of PDK/PDC. Overexpression of PDK1 in cancer cells inhibits the PDC-catalyzed tricarboxylic acid cycle (TCA cycle), resulting in the inability of aerobic oxidation, which may cause cancer cells to prefer other nutrients as their new energy source or other metabolic pathways to obtain large amounts of energy substances. Recent studies have suggested that the cancer-specific metabolic key enzyme such as PDCs [24] and PDKs [25] could be designed as potential therapeutic targets in diversified anticancer discovery efforts. Inhibition of PDK resulting in the activation of OXPHOS has turned out to be a feasible therapeutic strategy to reverse the Warburg effect and restrain cancer cell proliferation [26]. Downregulation of PDK1 suppressed the biological behavior of ovarian cancer cells due to S phase arrest and cellular apoptosis [27]. Reduced angiogenesis and increased necrosis in the OC316 and OVCAR3 tumor model were observed as the primary effect of PDK1 silencing in ovarian cancer [28].

Salt-inducible kinase 1–3 (SIK1-3) are serine/threonine kinases belonging to the AMP-activated protein kinase family which also play important roles in the regulation of HGSOC metabolisms [29]. SIK1-3 is highly expressed in 55% EOC and rarely detected in normal ovary tissues [30]. SIK2 could upregulate the transcription of major genes of glycolysis to enhance the Warburg effect of EOC through the PI3K-AKT-HIF1-α pathway [31]. Besides, SIK2 inhibits the mitochondrial OXPHOS through phosphorylation of DrP-1 at the Ser616 site [32]. Sirt3 is a sirtuin of NAD-dependent deacetylases in regulating reactive oxygen species (ROS) and ATP production [33, 34]. Sirt3 can reduce the stability of hypoxia-inducible factor 1α (HIF1α) and promote its degradation, resulting in the downregulating of glycolysis in cells [35]. Bcl2 inhibitor and STAT3 inhibitor cryptotanshinone could regulate the glucose metabolism and inhibit the growth of ovarian cancer cells through upregulating Sirt3-HIF1α [36].

Besides PDK/PDC and SIKs, hexokinase-2 (HK2) is another rate-limiting enzyme that catalyzes the production of glucose-6-phosphate **i**n the first step of glycolysis [37]. Pyruvate kinase M2 (PKM2) acts as another rate-limiting enzyme in the glycolysis process, and its main function is to catalyze the production of pyruvate [38–40]. Pyruvate is then reduced to lactic acid in a process catalyzed by lactate dehydrogenase A (LDHA) [41]. Changing the expression levels of several key proteins, including glucose transporter 1 (GLUT1) and rate-limiting enzymes in the glycolysis pathways (HK2, PKM2, and LDHA) could somewhat weaken the macromolecular synthesis and reverse the Warburg effect in ovarian cancer [42, 43].

### Accelerated glucose uptake in HGSOC

Augmented glycolysis and increased uptake of glucose are significant metabolic alterations of EOC cells [44, 45]. Recent studies showed that EOC cells can be categorized into glucose-deprivation sensitive (glucose addicted) and glucose-deprivation resistant (glucose non-addicted) based on their in vitro viability under glucose starvation. EOC patients with a glucose-addicted phenotype have significantly better progression-free survival than glucose non-addicted patients [46]. Metabolism reprogramming of glucose includes active glucose uptake, increased aerobic glycolysis, and decreased OXPHOS [47]. GLUT1 is a kind of membrane protein that primarily facilitates the transport of glucose into cells, and also provides additional glucose for energy metabolism [48].

Genomic landscape and cell signaling were considered key drivers of cancer cell metabolism. The genomic rewiring ranges from oncogenic mutations, and key kinases to signaling pathways on nutrients sensing, uptake/acquisition, and biosynthesis in tumor cells. Activation of oncogenes and loss of tumor suppressors promote metabolic reprogramming [49], resulting in a metabolism mode different from cells. The signaling mediators of glucose metabolism in ovarian cancer include PI3K/Akt, PTEN, MYC, and HIF1α [50, 51]. In normal cells, growth factor signaling activated RTKs-PI3K-AKT which are kinase-mediated signaling events, including the activation of receptor tyrosine kinases (RTKs) and the downstream phosphoinositide 3-kinase (PI3K) and AKT signaling cascade. The activation of AKT promotes expression and plasma membrane localization of the GLUT1. In cancer cells, glucose uptake is a cell-autonomously process through the activation of oncogenes not by stimulating growth factor signaling. And genetic alterations of the RTK encoding family increase their kinase activities. PIK3CA gene, encoding the catalytic subunit of PI3K, is one of the most frequently mutated genes in cancer. The metabolic function of the RTK family is also rewired for mutation of upstream regulating factors. HIF-α regulates glucose metabolism by inducing the VEGF-RTKS pathway in ovarian cancer. PDK1 is upregulated by HIF1α through the HIF1α-RTK pathway in ovarian cancer [52]. PTEN is a negative regulator of PI3K signaling and its loss-of-function mutations increase the downstream AKT signaling cascade.

### De novo lipogenesis in ovarian cancer

Alterations of lipid metabolism are critical to the proliferation and metastasis of ovarian cancer cells (see Fig. 1B).β-oxidation of fatty acids (FAs) consumes less oxygen than glucose which is a supplement of aerobic glycolysis [53]. Rapidly proliferating cancer cells have an increased demand for FAs for the construction of cell membranes, the formation of signaling molecules, and energy support [54]. There are two sources of FAs for the highly proliferating ovarian cancer cells: exogenous lipid uptake and endogenous de novo synthesis [55]. Large or rapidly growing ovarian cancers are abundant in hypo-vascular or hypoxic regions where the exogenous supply of FAs is scarce [56, 57]. Cancer cells rely on fatty acid synthase (FASN)-mediated de novo lipogenesis, despite their access to environmental lipids.

The de novo lipogenesis of FAs relies on the intermediates of glucose and amino acids. Cytosolic citrate resulting from glutamine metabolism and the citric acid cycle turns into a series of saturated FAs through ATP-citrate lyase [58], Acetyl-CoA carboxylase, and fatty acid synthase [59]. These initially generated FAs are next transformed into palmitoleic and oleic acid-mediated by the fatty acid elongases [60] and stearoyl CoA desaturases (SCDs) [61, 62]. Palmitoleic and oleic acids are further reduced into polyunsaturated FAs by other fatty acid desaturases. The majority of those fatty acid species are esterified with glycerol into triglycerides and stored in lipid droplets for further utilization [63]. Fatty acid desaturation is essential for the maintenance of membrane fluidity, cellular signaling, and providing energy through oxidation [64]. FAs required for tumor growth and proliferation are mainly derived from de novo synthesis, but normal cells tend to absorb exogenous FAs owing to the inhibition of de novo synthesis [65]. Orlistat, a pancreatic lipase inhibitor, acts as an irreversible inhibitor of FASN and has been shown to reduce proliferation and promote apoptosis in OC cells [66]. Therefore, inhibiting the endogenous fatty acid synthesis pathways of tumor cells becomes a potential anti-tumor treatment target.

Enzymes in the fatty acid synthesis pathway are also found to have increased expression levels across various cancer types and this increased expression correlates with worse survival outcomes. SCD1 is the rate-limiting enzyme converting saturated FAs to unsaturated FAs and is upregulated in various cancers [67]. Unsaturated FAs were enriched and essential for the proliferation and survival of stem cells in ovarian cancer [68]. Inhibition of SCD1 activity eliminated stem cells and retarded tumor initiation in ovarian cancer. Also, some types of EOC employ alternative means to acquire unsaturated FAs and therefore become less dependent on SCD1.

### The sensation and uptake of amino acids in EOC

Amino acids are of utmost necessity for cancer cell proliferation. Proliferating cancer cells often engage in amino acid production more proactively and biosynthesis of non-essential amino acids (NEAAs) (see Fig. 1C). Active intake of amino acids and macropinocytosis scavenging of proteins are two crucial amino acid sources to support cell proliferation [69, 70].

Tumor cells rewired their metabolic program to actively acquire amino acids from the extracellular space to sustain biomass accumulation. The mechanistic target of rapamycin complex 1 (mTORC1) is a central coordinator of amino acid availability and allocation [71]. Its activity is tightly controlled by the growth factor signaling inputs and the availability of amino acids. mTORC1 is localized in the proximity of ras homolog enriched in the brain (RHEB) in the lysosome [72]. Tuberous sclerosis complex subunit 2 (TSC2) represses the activation of mTORC1 through binding to the lysosomal-resident target of RHEB [73]. Amino acid levels regulate the activity of mTORC1 through specific amino acid sensor proteins and downstream RAG GTPases which inhibit mTORC1 by promoting its localization to the proximity of RHEB [73]. The intracellular amino acid sensors directly influence amino acid consumption by decreasing mTORC1 stimulation of translation when these amino acids become limiting [74, 75]. Both CASTOR1 and SAMTOR function as negative regulators of the RAG GTPases through the GATOR2–GATOR1 axis. When the cellular leucine level decreases, Sestrin2, a leucine sensor, can bind and inhibit GATOR2, leading to the activation of the GATOR1 complex that represses the RAG GTPases and mTORC1 activation [73]. CASTOR1 was identified as a sensor of cytosolic arginine [74], and SAMTOR was shown to be a sensor of S-adenosyl methionine and hence indicative of the cellular methionine level [75].

Besides the active uptake of amino acids, cells can specifically adjust the levels of certain amino acids by modulating the expression of selective transporters and/or by regulating de novo biosynthetic pathways. When PI3K -AKT signaling is activated, TSC2 is dislocated from RHEB and results in the activation of mTORC1 kinase activity. Concerted AKT and mTORC1 kinase activation induce and sustain cell surface expression of amino acid transporters [76]. mTORC1 activation increases the protein synthesis largely through direct phosphorylation of p70S6 kinase 1(S6K1) and eIF4E binding protein 1 (4EBP1) [71]. Also, through S6K1 kinase activity, mTORC1 can induce ribosome biogenesis to sustain the expanded protein translation capacity [77].

Macropinocytic scavenging of proteins has been demonstrated to be a crucial opportunistic amino acid source to support cell proliferation, especially in cancer. The initiation of macropinocytosis is characterized by membrane ruffling and macropinosome formation and is normally stimulated by growth factor signaling and PI3K activation [78, 79]. Oncogenic Ras is also a major regulator of the rate and volume of macropinosome uptake. Macropinosome cargo can sustain the proliferation of RAS mutant cancer cells by providing nutrients in poorly vascularized tumor regions [80–82]. As part of the cellular nutrient acquisition network, macropinocytosis is coordinated with other nutrient sensing and nutrient responsive pathways in a concerted manner. The AMPK monitors energy stress by sensing an increase in AMP to ATP ratio, and via its kinase activity, restores metabolic homeostasis by suppressing anabolism and enhancing catabolic processes such as macroautophagy. As a master sensor of certain amino acids, mTORC1 acts as a negative regulator of macropinocytosis trafficking to the lysosome. Suppression of mTORC1 activity limits the utilization of extracellular free amino acids, but greatly enhances the catabolism of extracellular proteins via micropinocytosis [82–85]. This paradoxical role of mTORC1 may explain the fact that while genes encoding RTK, PI3K, and PTEN are most frequently altered in many types of cancer, activating mutations in mTORC1 are rare. Constitutive mTORC1 activation would lead to increased dependencies on the utilization of extracellular free amino acids and hence would limit the flexibility of nutrient acquisition strategies in the face of fluctuations in the extracellular environment during tumor progression.

### De novo biosynthetic pathway of NEAA in EOC

Except for amino acid uptake, tumors are capable of synthesizing NEAAs with the metabolites of glycolysis and the TCA cycle. When cells sense a decrease in amino acid levels through general control nonderepressible 2 (GCN2), activating transcription factor 4(ATF4) can directly lead to the upregulation of enzymes involved in NEAA synthesis, such as asparagine synthetase (ASNS) in asparagine synthesis [86], and phosphoglycerate dehydrogenase (PHGDH) and phosphoserine aminotransferase 1 (PSAT1) in serine biosynthesis [87].

Aberrant glutamine metabolism, with overexpression of glutaminase (GLS), was associated with poor survival in EOC patients and platinum resistance in EOC cellular models [88]. Alterations of several metabolic pathways, such as histidine and tryptophan metabolism, arginine biosynthesis, arginine and proline metabolism, and alanine, aspartate, and glutamine metabolism, were found in the sera of EOC patients [89]. Moreover, metabolic analysis of 98 plasma samples defined kynurenine, acetyl-carnitine, phosphatidylcholine, and lysophosphatidylethanolamine as potential predictive biomarkers to distinguish short-term from long-term EOC survivors [90].

The multi-omics studies of glycoproteome, transcriptome, and proteome of HGSOC have revealed that the glycans conjugated on glycosylation sites are associated with tumor subtypes and intact glycopeptides information from a combination of glycosylation sites and site-specific glycosylation provide survival predictors beyond the protein and transcription levels, which provide significant clues for the treatment of HGSOC [91].

### Materials supplication from autophagy in EOC

**Fig. 2:**
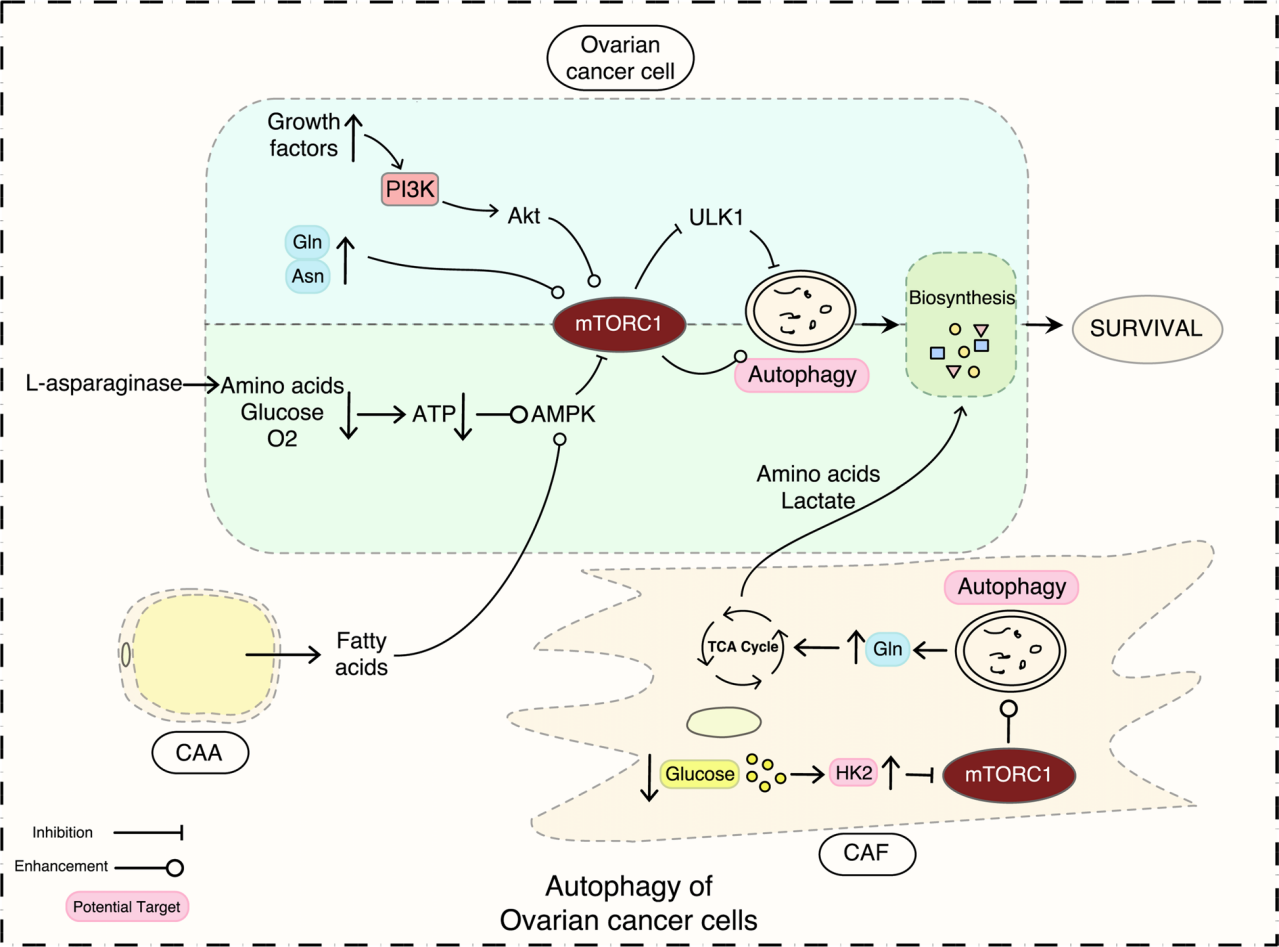
The role of autophagy in ovarian cancer cell metabolism. CAA, cancer associated adipocyte; CAF, cancer-associated fibroblast

As the proliferation rate of ovarian cancer is fast, the NEAA acids and FAs provided from external sources for the endogenous synthesis do not meet the increased needs of the highly proliferating cancer cells [55]. However, the TME is characterized as nutrient-deprived and energy-limited. Under the extra- and intracellular regulation, autophagy is triggered and sustains cancer cell survival by producing metabolites that can be re-used in biosynthetic processes or energy production [92]. mTORC1 complex is the main negative controller of autophagy. The absence of nutrients (amino acids, glucose) and the lack of oxygen leads to reduced production of ATP and further triggers autophagy through the AMPK pathway. Reversely, the abundant presence of amino acids could directly or indirectly activate mTOR via the growth factors-PI3KC1–AKT pathway to inhibit cell autophagy [93, 94] (see Fig. 2).

Prolonged amino acid starvation in ovarian cancer cells can be sensed by the mTOR kinase and triggers the expression of several autophagy genes [95]. Ovarian cancer cells are particularly addicted to several essential amino acids, such as glutamine and arginine [96]. The lack of essential amino acids determines the proteasomal degradation of the mTOR kinase and allows pro-survival autophagy [97]. The nutrient addiction of EOC cells relies both on the availability of different substrates and the cancer stage [98, 99]. Interestingly, in high-invasive EOC cells, glutamine fuels the cancer cells through the Krebs’s cycle when glucose is not fully oxidized in the mitochondria [96] while glucose sustains the energetic and anabolic processes as the main sources in low-grade EOC cells, which are glutamine-independent [100]. Cancer cells induce surrounding cancer-associated fibroblasts (CAFs) to synthesize more glutamine. Glutamine has been shown to stimulate EOC cell proliferation through modulating the mTOR pathway [100] which can be inhibited by L-asparaginase. The latter can degrade glutamine to glutamate and induce autophagy in EOC cells [46]. When inhibiting the endogenous synthesizing and depriving exogenous sources of arginine, the ovarian cancer cells lose the function to induce autophagy and eventually fall deathward [101].

Cancer cells located in hypoxic niches use aerobic glycolysis for energy supply [102]. Aerobic glycolysis consumes a huge amount of glucose for less convenience than Kreb's cycle [103]. The lack of glucose, one of the principal sources of energy, is sensed by the cancer cells through the HK2-mTOR axis, which triggers autophagy as a stress response [104]. However, glucose-deprived induced autophagy occurs mainly in CAFs, not in cancer cells [105]. Then the CAFs supply the cancer cells with amino acids and lactate to sustain the cancer progression [106, 107]. During energy stress conditions, cancer-associated adipocytes (CAAs) upregulate autophagy by the activation of the AMPK-mTOR axis and supply FAs for cell membrane construction or β-oxidation [108]. Metastatic EOC in omentum may rely on FAs to fuel the high bioenergetic demand of cancer cells instead of glucose [109, 110] through the mTOR-dependent activation of myeloid-derived suppressor cells [109].

### Nutrients and metabolite composition regulation

Tumor cells also reprogram the surrounding stromal cells by paracrine growth factors, cytokines, and other signaling molecules, such as platelet-derived growth factors and VEGF, to create a favorable environment for continuous proliferation. Besides, the levels of major carbon and nitrogen sources such as glucose, and glutamine affect their metabolic reprogramming and metabolic phenotype. In low glucose conditions, tumor cells could not maintain cellular ATP levels after metformin blocks mitochondrial oxidative metabolism [111]. When glucose increases, the tumor cells become less responsive to metformin which may be contributed to activated glycolysis and lactate recycling in high glucose concentrations. Within the same organ, solid tumors themselves are metabolically heterogeneous. Previous studies have indicated that cancer cells in highly perfused areas consume glucose to sustain glycolysis and OXPHOS, while cells in lowly perfused areas rely on other carbon sources [112]. Interestingly, these metabolic preferences were found to be dependent on different oncogenic drivers [113]. The antiporter xCT/SLC7A11 mediates the transport of cystine and glutamate across the plasma membrane. The import of cystine allows for glutamate secretion and glutamine catabolism [114]. Then, low levels of cystine make cancer cells less susceptible to glutamine inhibitors [115] which seems to sustain OXPHOS more dependent on mitochondria glucose-dependent PDK and PDC activity in Ras-driven cancer [113].

Enzymatic activity involving metabolism is delicately balanced and regulated. Besides upstream regulation, the compartmentalization of competing reactions into different membrane-bound organelles, also called Liquid–liquid phase separation (LLPS), is another important regulation. LLPS is a dynamic and reversible process that includes two distinct phases, the dense and dilute phase [116, 117]. The dense phase enriched in condensed biomolecules often takes the form of large droplets whereas the surrounding diluted phase is depleted of those components. The LLPS-related gene signature was an independent prognostic factor of ovarian cancer. However, more studies are needed on the relationship between metabolic enzymes and LLPS formation [118].

## Invasion and metastasis of EOC

**Fig. 3:**
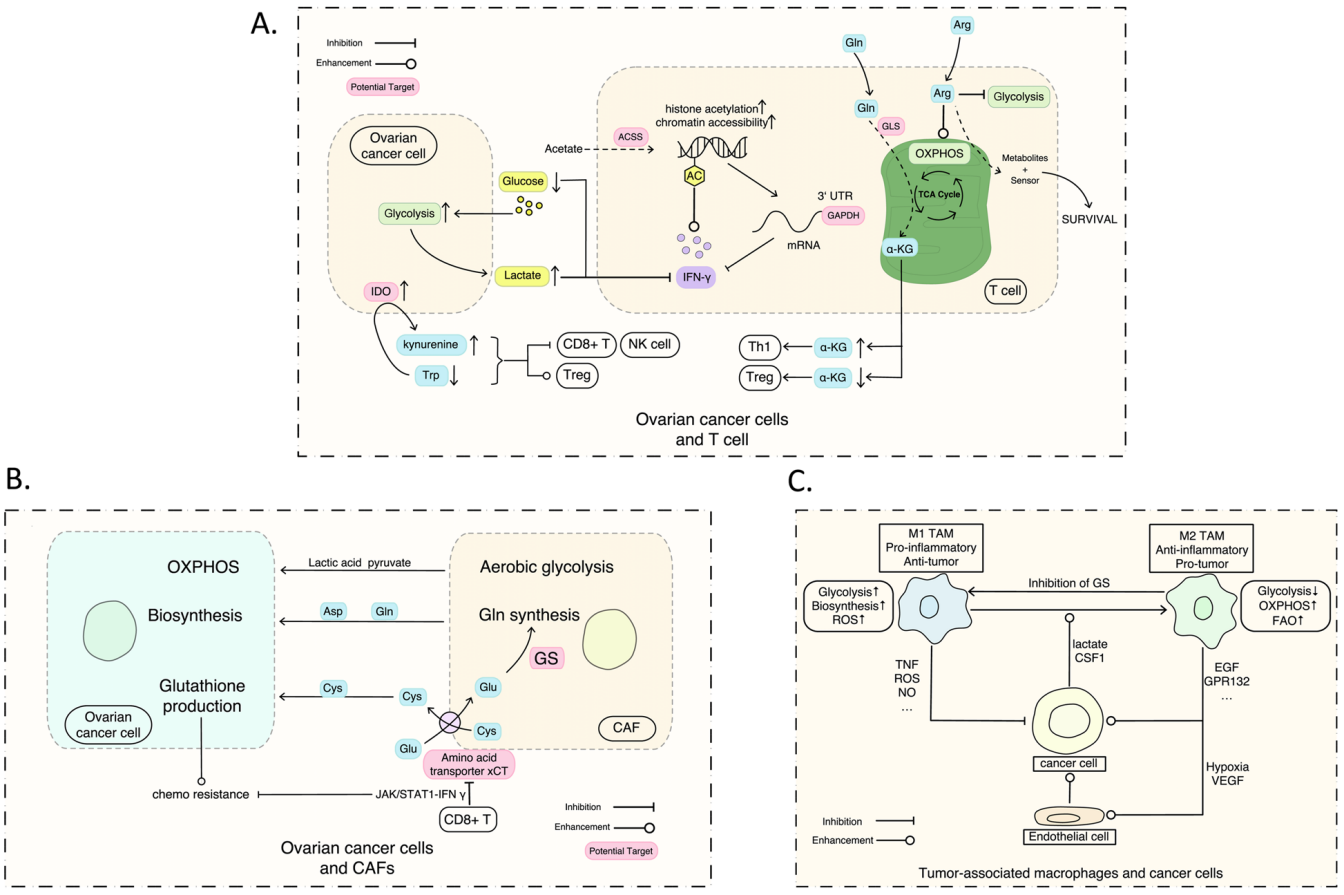
The interaction between ovarian cancer cells and immune cells of TME. **A** ovarian cancers compete with T cells of TME; **B** CAFs promote the metabolism of ovarian cancer cells; **C** shows dual relationships between the ovarian cancer cells and TAMs. TME, tumor microenvironment; CAFs, cancer-associated fibroblasts; GS, glutamine synthetase; TAMs, tumor-associated macrophages

Activating invasion and metastasis and avoiding immune destruction compromised the basis of EOC invasion and metastasis. Omentum and peritoneum implantation is the hallmark of advanced-stage EOC. Tumor growth and metastasis require a special microenvironment [119], commonly including immune cells (macrophages, neutrophils, eosinophils, T cells, B cells), stromal cells (adipocytes, fibroblasts, vascular endothelial cells), extracellular matrix (ECM) (proteoglycan, hyaluronic acid, collagen, fibronectin, laminin), and soluble factors(growth factors, chemokines, cytokines, and metabolites) [120]. The metabolic landscape of ovarian cancer cells is shaped by its unique niche which is hypoxic, acidic, nutrient-deprived, and characterized by electrolyte imbalance and elevated oxidative stress [112, 121–123]. The tumor cells are inhabited in the TME and struggle for an advantage over non-cancerous cells, dysfunctional blood flow, and increased inflammation to satisfy its high metabolic activity. The altered non-cancer cells and ECM components of TME also contribute to the cancer metabolism and behavior which show potential metabolic crosstalk or competition within the TME (see Fig. 3).

### Alteration and interaction with the stromal cells of ovarian cancer

Tumor-associated stromal cells include different cell types: CAFs, CAAs, or cancer-associated endothelial cells [124]. Contrary to immune cells, epithelial cancer cells can reprogram the catabolism of neighboring stromal cells to enhance their secretion of energy-rich metabolites (such as pyruvate, lactate, amino acids, and free FAs) that are up-taken by tumor cells to sustain their bioactivities [105, 125, 126].

#### CAAs

Omentum is comprised of adipocytes which are the preferred metastatic site of ovarian cancer cells [127]. CAAs release FAs which are the energetic sources of ovarian cancer. FAs are translocated across the phospholipid bilayers of the plasma membrane through either passive diffusion or a saturable protein-mediated transport system. Several membranes associated with FA binding proteins and transporters reportedly facilitate the transport process, including FA translocase CD36, fatty acid transport proteins (FATPs), and fatty acid-binding proteins (FABP). Lipids are imported into cells through a variety of fatty acid transporters which include low-density lipoprotein receptors, FATPs, fatty acid translocase, and FABPs [128, 129]. Adipocytes are the key carriers for ovarian cancer cells to enter the omentum. When cultured with primary adipocytes, ovarian cancer cells rely more on the uptake of exogenous lipids and cholesterol than on de novo lipogenesis. Fatty acid translocase CD36 is the predominant fatty acid transporter in cancer cells. Primary adipocytes could induce ovarian cancer cells to express CD36 which enhances the fatty acid uptake and lipid droplet accumulation of cancer cells. Increasing exogenous fatty acid uptake could further reduce endogenous lipid metabolism and cholesterol biosynthesis. Knockdown of CD36 suppressed the adipocyte-induced cellular invasion and metastasis in both in vivo and vitro assays [130]. Furthermore, ovarian cancer cells were shown to consume arginine secreted by the adipose stromal cells and convert it to citrulline and nitric oxide (NO). While citrulline enhances adipogenesis in adipose stromal cells, NO reduces oxidative stress and promotes glycolysis in cancer cells [131]. The adipokines stimulate cancer progression [132]. When cultured in the ascites microenvironment, ovarian cancer cells' metabolism modality shifts from aerobic glycolysis to β-oxidation and lipogenesis which is associated with increased aggressiveness [133].

FABP4 was a lipid chaperone protein in EOC cells and also highly upregulated in metastatic EOC cells. FABP4 interacts with the transmembrane domain of CD36 to facilitate the import of free FAs from the extracellular space. Once inside the cell, FAs remain bound to the cytosolic part of FABPs until their delivery to the destination site. FABP4 promotes the colonization of tumor cells in the lipid-rich TME, and its targeted inhibitor inhibits the adaptation of tumor cells to colonization in the TME [134]. FABP4 also mediates the direct transfer of lipids between adipocytes and EOC cells. Knockdown of FABP4 could diminish the metastatic potential of HGSOC cells. Tamoxifen, an inhibitor of FABP4, could impair the uptake of free FAs and inhibit EOC cell migration and invasion [135]. BMS309403, a small molecule inhibitor of FABP4, could not only significantly reduce tumor burden in a syngeneic orthotopic model but also increase the sensitivity of cancer cells towards carboplatin [136].

#### CAFs

The peritoneum is another preferred metastatic site of ovarian cancer cells compromised with fibroblast cells. Further studies have found that the Warburg effect of the tumor occurs in the surrounding myofibroblasts, rather than in the tumor cells themselves. Tumor cells can induce the surrounding CAFs to proceed with aerobic glycolysis through which these CAFs generate high energetic metabolites (such as lactic acid and pyruvic acid). Those metabolites can be taken up by adjacent tumor cells for further mitochondrial OXPHOS which plays a crucial role in tumor genesis, invasion, and metastasis through the systemic interaction of energy metabolism with the tumor at all stages of its evolution [126]. CAFs are also characterized by an increased glutamine anabolic metabolism. Glutamine gets secreted in the TME and is consumed by cancer cells to sustain nucleotide generation and OXPHOS. Notably, the combined inhibition of glutamine synthetase (GS) in CAFs and GLS in tumor cells decreased tumor growth and metastasis in an ovarian cancer mouse model [137]. Besides, CAFs can also secrete aspartate, which promotes nucleotide biosynthesis and proliferation in ovarian cancer. The metabolic exchange between CAFs and tumor cells is bidirectional. CAFs provide cysteine to cancer cells for glutathione production, conferring resistance toward platinum-based chemotherapeutic drugs. Notably, CD8 + T cells abrogate this resistance through interferon-gamma (IFNγ) production, which represses the cystine-glutamate antiporter (xCT) in CAFs via JAK/STAT1 signaling [138].

### Microenvironment ovarian cancer metabolism

#### T cells

The immune cells of TME include the innate immune response cells (such as macrophages, and natural killer cells) and the adaptive immune response cells (such as CD4 + T cells and CD8 + T cells). Nature killer cells and CD8 + T cells are classified as cytotoxic lymphocytes which offer a natural defense against tumor progression through the specific killing of tumor cells after identifying the tumor antigens. CD4 + T cells support (Th1, Th17) or repress (Treg) the activity of cytotoxic lymphocytes. When T cells interact with an antigen and induce signaling through the T cell receptor (TCR), a cascade of signaling events is initiated that trigger rapid metabolic remodeling [139]. The function of T cells relies on their metabolism and the metabolic alterations affect their fate.

Highly proliferative activated T cells and cancer cells both rely heavily on glucose metabolism and compete for energy nutrients like glucose and amino acids. Cancer cells can inhibit the CD8 + T cell infiltration and proliferation through several mechanisms [140]. Glycolysis is important to promote T cell effector function by sustaining IFNγ production. Glucose deprivations inhibit transcription of IFN through binding the AU-rich region in the 3’ UTR of cytokine mRNA with GAPDH enzymes, reducing mTOR activity, and accumulation of the glycolytic product lactate. Previous studies demonstrate that metabolic targets can be utilized to rescue T cell function in a metabolically hostile environment. Despite the importance of glucose and lactate levels, T cells appear to exhibit some degree of metabolic flexibility. Acetate can rescue IFNγ production in glucose-restricted T cells by promoting histone acetylation and chromatin accessibility in an acetyl-coenzyme A synthetase (ACSS)-dependent manner [141]. Additionally, CD8 + T cells upregulate fatty acid catabolism to provide energy to preserve their effector function [142].

Aside from glucose, amino acids have now also been identified to drive and fuel T cell function and differentiation [143, 144]. As described for glucose, arginine uptake and catabolism of immune cells have also been shown to be outcompeted by ovarian cancer cells [145]. The balance of this competition has been linked to the expression and activity of amino acid transporters and metabolic enzymes that act as rate-limiting factors for metabolite uptake and conversion [146]. Specific clones of human ovarian cancer cells show high levels of expression of the enzyme indoleamine 2,3-dioxygenase (IDO) which is important for the catabolism of tryptophan [147]. IDO activity boosts the decomposition of tryptophan and generates tryptophan-derived catabolites, such as kynurenine. Both the scarcity of tryptophan and the accumulation of kynurenine have been found to markedly suppress the proliferation and killer function of activated CD8 + T-cells and NK cells, and promote a Treg phenotype [148, 149]. Because cancer cells seem to outcompete immune cells in ovarian cancer models, potentiating immune cell function through modulation of amino acid catabolism represents a unique opportunity to shift this balance.

#### Macrophages and tumor cells

Like T cells, tumor-associated macrophages (TAMs) also compete with ovarian cancer cells for glucose. However, the TAMs have two phenotypes that play an opposite function in tumor growth. M2 macrophages have a pro-tumoral function through secretion of growth-promoting cytokines and local immunosuppression, whereas M1 macrophages can directly limit primary tumor growth [150].

Several factors regulate the polarization of TAMs towards an M1 or M2 phenotype: genetic background, surrounding soluble factors (such as cytokines, and chemokines), and cell metabolism [151–153]. Glycolytic activity in TAMs has mainly been associated with tumor regression. REDD1 is the negative regulator of mTOR and further decreases the activity of glycolysis. Hypoxic TAMs have increased the expression of REDD1 in M2 TAMs [150]. After the knockout of REDD1, TAMs outcompete endothelial cells for glucose utilization to promote tumor vessel normalization and impair metastatic spread [154].

Lactate produced by tumor cells has a critical function in signaling and TAM polarization. Specifically, lactate induces a pro-tumoral M2 phenotype by inducing VEGF through HIF1α stabilization [155] and through activation of the G protein-coupled receptor 132 to enhance cancer metastasis [156]. Glutamine metabolism promotes fatty acids oxidation and epigenetic activation of M2 genes [157]. In starvation conditions, the expression and activity of GS are also increased in the M2 TAMs. Inhibiting the activity of GS promotes a switch from an M2 to an M1 phenotype and ultimately prevents tumor cell spread. The ascites of ovarian cancer present high concentrations of linoleic acid which can activate PPARb/d and further induce the pro-tumorigenic polarization of ovarian TAMs [158].

## Metabolic evolve of EOC to the stress of treatment

The reaction of cancer cells after treatment remains different. Cancer cells may adapt to the different metabolic modes when undergoing metabolically challenges.

### Chemotherapy of paclitaxel and platinum

The relapse of EOC is mainly attributable to late diagnosis and the development of chemoresistance [159]. Increasing in glucose and glutamine metabolism following platinum treatment was observed in EOC models, suggesting that tumor cells under the pressure of drug treatment may further reprogram their metabolism to improve their fitness and survival capability [160]. EOC cells can be divided into glucose-deprivation sensitive and glucose-deprivation resistant based on their in vitro viability under glucose starvation. Patients with a glucose-deprivation sensitive phenotype are frequently associated with alterations of the p53 and PI3K/Akt/mTOR pathways that are commonly detected in EOCs, and that are known to be associated with chemotherapy resistance [161].

The pentose phosphate pathway (PPP) is elevated in tumor cells. PPP usually occurs in the cytoplasm. The intermediates of PPP are phosphate, including NADPH, pentose phosphate, and fructose 6-phosphate. NADPH can reduce the level of active oxides, like free radicals, caused by radiotherapy and chemotherapy, maintain redox homeostasis, and protect tumor cells from oxidative damage. Pentose phosphate is involved in ribonucleotide biosynthesis. Moreover, PPP is also involved in fatty acid synthesis and glycolysis [162]. Activation of PI3K/Akt, Ras, and Src signaling pathways in various malignant tumor cells upregulates the expression of PPP-related protein, leading to radiation and chemotherapy resistance [163].

Ovarian cancer cells also produce high levels of ROS, likely due to defective signaling pathways. Mitochondria-associated granulocyte colony-stimulating factor stimulating protein (Magmas) is a ROS scavenger, that is also overexpressed in ovarian cancer cells. Magmas inhibitor was able to sensitize an ovarian cancer cell line to carboplatin [164].

### PARP inhibitors

In a study with-depth, single-cell phenotypic characterization of HGSOC, patients with poorer outcomes had an increased frequency of cell type that co-expressed vimentin, HE4, and c-Myc. Low c-Myc/HE4 expressed cells were sensitive to continuous exposure to carboplatin. However, cells with a high c-Myc/ HE4 expressed subgroup were sensitive to c-PARP treatment, not carboplatin [165]. These HGSOC patients may be good candidates for BrD4 inhibitors which are small molecules known to disrupt c-Myc function [166]. Similar to SIRT proteins, the PARP family is also an NAD + dependent enzyme that targets proteins using nicotinamide adenine dinucleotide (NAD +) as a donor [167, 168]. SIRT1 and PARP1 are both NAD + dependent, these enzymes compete for the NAD + pool [169]. Observations indicate that the decline of NAD + consumption by PARP1 activity correlates with a down-regulation in SIRT activity. Once DNA is damaged, PARP activation depletes cellular NAD + pools, hampering cellular energy metabolism by reducing SIRT1 activity [170]. Combination treatment targets both PARP and Sirt inhibitors may result in a coordination effect.

### VEGF inhibitors

One of the targeted therapies for EOC is anti-angiogenic therapy. One significant characteristic of ovarian cancer is angiogenesis induced by low oxygen. In ovarian cancer xenografts, a remarkable alteration occurred in tumor lipidomic profile after the anti-VEGF treatment with bevacizumab, including increased levels of triacylglycerols and reduced saturation of lipid chains. Further transcriptome analysis revealed up-regulated metabolism pathways accompanied by increasing accumulation of lipid droplets both in vitro and in vivo assays. After treatment with liver x-receptor agonists GW3965, this phenomenon could be selectively counteracted which resulted in the therapeutic effects of bevacizumab being enhanced and the viability of tumor cells inhibited [171].

### PD-L1 inhibitors

GLS is an enzyme that converts the amino acid glutamine into glutamate. ARID1A is a part of the SWI/SNF protein complex, which inhibits the widely expressed GLS gene. Inactivation of ARID1A increases glutamine utilization and metabolism through the TCA cycle to support aspartate synthesis. In ovarian clear cell carcinoma (OCCC), GLS inhibitor CB-839 suppresses the growth of the tumor with ARID1A mutation, but not wildtype, and significantly reduces the tumor burden and prolongs the survival in patient-derived xenografts. In addition, glutamine metabolism regulates the function of immune lymphocytes in the TME. The glutamine antagonism in effector T cells can be used as a "metabolism checkpoint". Hence, "metabolism checkpoint blockade" GLS inhibitor CB-839 could further enhance the therapeutic effectiveness of immune checkpoint inhibition anti-PD-L1 [172].

## Targeting treatment of ovarian cancer based on metabolism

Targeting cancer cell metabolism has been an attractive therapeutic target in various cancers. In ovarian cancer, targeting the metabolism of cancer stem cells through inhibition of lipid metabolism resulted in the elimination of cancer stem cells and decreased tumor development in mouse models. However, targeting cancer cell metabolism in the clinic has been largely unsuccessful either due to a lack of efficacy or safety [173]. This is likely due to a lack of specificity of the small molecule inhibitors. One therapeutic that has shown the potential to provide clinical benefit is metformin. Besides effects on metabolism, potential mechanisms also include inhibition of the epithelial to mesenchymal transition, AMPK signaling, and apoptosis induction [174–176]. Preclinical models in ovarian cancer demonstrated the anticancer effect [177, 178]. Clinical studies have shown that metformin can affect ovarian cancer stem cells and the tumor stroma [179]. Currently, metformin is evaluated as a single agent before surgical debulking and combined with chemotherapy in the treatment of ovarian cancer (NCT03378297, NCT02437812).

Based on the metabolic characteristics of ovarian cancer cells, multiple pathways may be the target of future ovarian cancer therapy. First, adjust the dietary structure to reduce tumor-specific raw nutrient intake. For example, removal of serine through dietary changes or pharmacological pathways shows potential in P53 deficient tumors. De novo serine synthesis appears essential to cancer cells with PHGDH overexpression. Inhibition of the de novo synthesis pathways of FAs and amino acids (such as serine, and glutamine) is important. Cancer cells may actively adapt to the utilization of varieties of surrounding nutrients, particularly during metastasis as they invade new territories that are metabolically challenging. Nutrient uptake and utilization are direct results of extracellular stimuli and maintaining glucose influx and metabolism are essential to sustain cell survival and growth. Targeted drugs inhibit the uptake of specific sugars, FAs, and amino acids through selective inhibition of their transporters.

Besides, proliferation exceeds the ability of the existing vasculature to supply sufficient oxygen and nutrients when in nutrient and energy crises. Also, autophagy is a critical catabolic process whereby cells salvage intracellular constituents under the conditions of nutrient scarcity. The treatment of cells in rapid proliferation needs the combination of several targets, such as the combination of anti-angiogenesis and elements starvation with anti-mTORC1.

In the last, the sense and regulation of metabolism also involve several balances, such as ROS, ratios of ATP/ADP, nutrient supply, and microenvironment adaptation. The first balance is ROS (KEAP1-NRF2 axis) which is regulated by NADH-NAD + balance and PI3K. Local high NADH to NAD + ratio, thioredoxin system, and the glutathione system, reducing equivalent of NADPH to NADP + , therefore contributing to cellular redox balance by serving as an electron acceptor and increasing the ROS level, can stimulate cell growth and inhibit cancer metastasis. Next is the ratio of ATP/ADP, when cells rapidly undergo apoptosis as the reduction in mitochondrial membrane potential and cellular ATP level. Opportunistic modes of nutrient acquisition from the TME meet the needs of cancer cells proliferation and invasion. Interruption of the above balances is also the underlying treatment target of ovarian cancer.

## Conclusion

The metabolism alterations involve the proliferation, metastasis, and treatment resistance of epithelial ovarian cancer, especially high-grade serous ovarian cancer. EOC cells satisfy their rapid proliferation through the accelerated uptake of glucose, amino acids, and lipids. Ovarian cancer cells complete their implanted metastasis by utilizing the nutrients and interacting with other cells of the special microenvironment. Lastly, the cancer cells evolve through metabolism rewiring under the treatment stress of chemotherapy and target therapy. However, there are no systematic studies on metabolic homeostasis imbalance and metabolic remodeling during the development of EOC. Further studies are needed.

## Data Availability

All the data obtained and analyzed during the current study were available from the corresponding authors on reasonable request.

## Abbreviations

EOC: Epithelial ovarian cancer
VEGF: Vascular endothelial growth factor
PARPi: Poly ADP-ribose polymerase inhibitors
PD-1: Programmed cell death protein-1
PD-L1: Programmed cell death-Ligand 1
HGSOC: High-grade serous ovarian carcinoma
OXPHOS: Oxidative phosphorylation
TME: Tumor microenvironment
PDK: Pyruvate dehydrogenase kinase
PDC: Pyruvate dehydrogenase complex
SIK 1–3: Salt-inducible kinase 1–3
HIF1α: Hypoxia-inducible factor 1α
ROS: Reactive oxygen species
HK2: Hexokinase-2
PKM2: Pyruvate kinase M2
GLUT1: Glucose transporter 1
LDHA: Lactate dehydrogenase A
RTKs: Receptor tyrosine kinases
FAs: Fatty acids
PI3K: Phosphoinositide 3-kinase
FASN: Fatty acid synthase
SCDs: Stearoyl CoA desaturases
mTORC1: Mechanistic target of rapamycin complex 1
RHEB: Ras homolog enriched in brain
ASNS: Asparagine synthetase
PHGDH: Phosphoglycerate dehydrogenase
PHGDH: Phosphoserine aminotransferase 1
TSC2: Tuberous sclerosis complex subunit 2
NEAAs: Non-essential amino acids
TCA cycle: Tricarboxylic acid cycle
GCN2: General control nonderepressible 2
ATF4: Activating transcription factor 4
CAFs: Cancer-associated fibroblasts
GS: Glutamine synthetase
GLS: Glutaminase
CAAs: Cancer-associated adipocytes
LLPS: Liquid–liquid phase separation
ECM: Extracellular matrix
FATPs: Fatty acid transport proteins
FABPs: Fatty acid-binding proteins
IFNγ: Tumor-interferon-gamma
ACSS: Acetyl-coenzyme A synthetase
IDO: Indoleamine 2,3-dioxygenase
TAMs: Tumor-associated macrophages
FAO: Fatty acids oxidation
PPP: Pentose phosphate pathway
Magmas: Mitochondria-associated granulocyte colony-stimulating factor stimulating protein
NAD +: Nicotinamide adenine dinucleotide
OCCC: Ovarian clear cell carcinoma
